# Supplementary material for: Evaluation of Pharmacokinetic Feasibility of Febuxostat/L-pyroglutamic Acid Cocrystals in Rats and Mice
Source: Pharmaceutics. 2023 Aug 21;15(8):2167. doi: 10.3390/pharmaceutics15082167 (PMC10459842; doi:10.3390/pharmaceutics15082167)
Supplement: Supplementary file 1 [file pharmaceutics-15-02167-s001.zip › pharmaceutics-2551318-supplementary.pdf]

Supplementary material

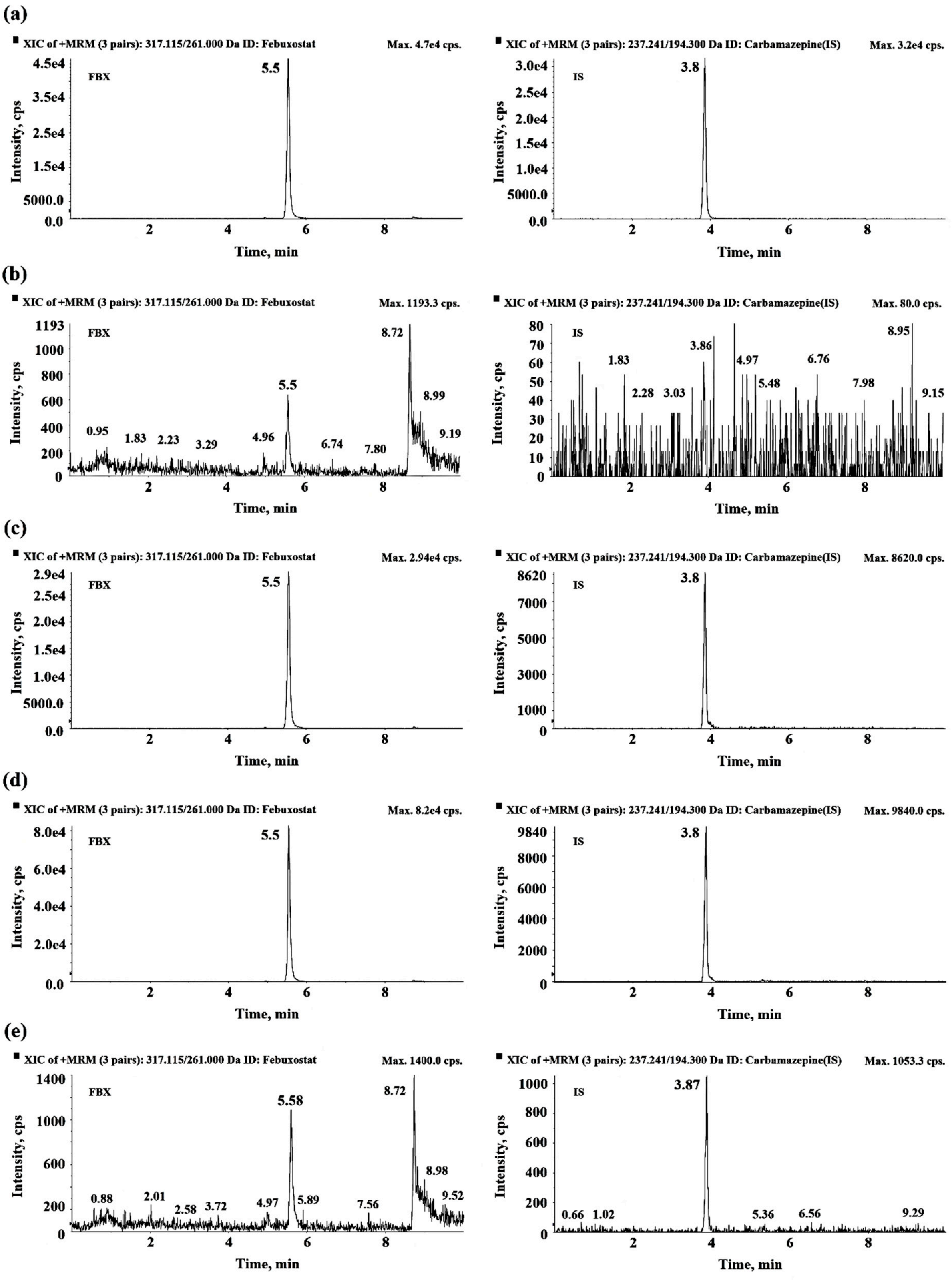

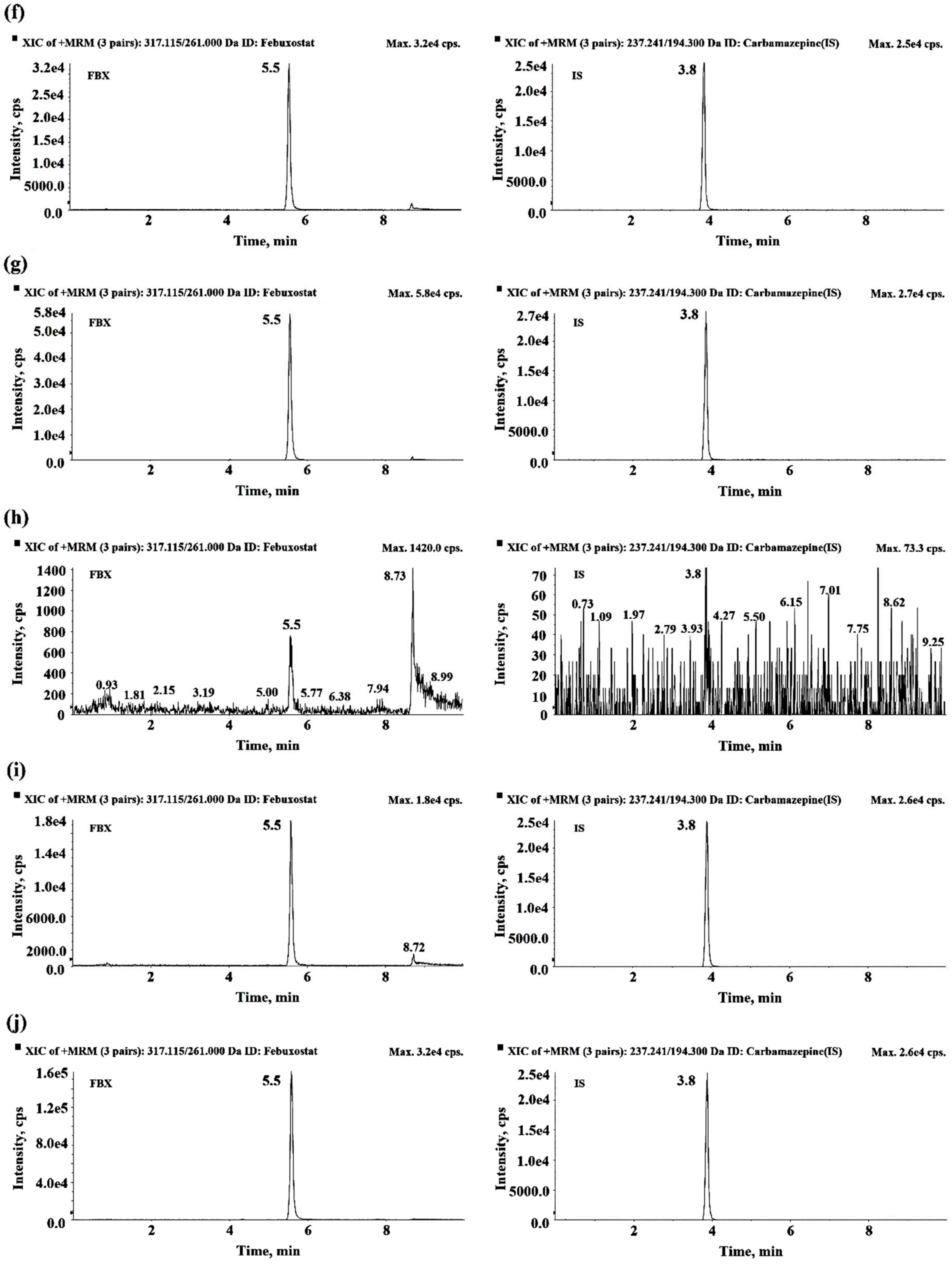

Figure S1. Representative chromatograms of FBX and IS in mouse plasma, urine, and GI samples. **(a)** Stock solution of 0.005 μg/mL FBX and 0.005 μg/mL IS; **(b)** drug-free mouse plasma; **(c)** plasma standard spiked with 7 μg/mL FBX; **(d)** plasma sample at 30 min after oral administration of 50 mg/kg FBX; **(e)** drug-free urine; **(f)** urine standard spiked with 7 μg/mL FBX; **(g)** urine sample collected at 24 h after oral administration of 50 mg/kg FBX; **(h)** drug-free GI; **(i)** GI standard spiked with 7 μg/mL FBX; **(j)** GI sample collected at 24 h after oral administration of 50 mg/kg FBX.

Table S1. Concentrations (μg/mL for plasma and μg/g tissue in tissues) of FBX in plasma and tissues and their T/P ratios after oral (50 mg/kg as FBX) administration of FBX or FBX-PG to mice, respectively. Values in parentheses are mean values of the T/P ratios.

Tissue	FBX (n = 6)				
	**0.5 h**	**2 h**	**4 h**	**8 h**	**12 h**
Plasma	22.4 ± 6.49	22.6 ± 7.76	11.8 ± 8.00	4.91 ± 1.28	11.4 ± 6.95
Liver	32.4 ± 4.76	37.7 ± 8.24	23.7 ± 10.1	21.3 ± 11.9	26.3 ± 15.1
	(1.53 ± 0.395)	(1.75 ± 0.431)	(2.43 ± 0.758)	(4.13 ± 1.32)	(2.40 ± 0.286)
Kidney	11.1 ± 3.85	11.3 ± 7.18	12.1 ± 9.80	8.64 ± 3.34	8.00 ± 5.43
	(0.512 ± 0.168)	(0.523 ± 0.363)	(0.952 ± 0.450)	(1.89 ± 1.00)	(0.662 ± 0.160)
Stomach	112 ± 52.8	189 ± 59.8	125 ± 56.8	75.4 ± 29.7	87.9 ± 55.0
	(5.55 ± 2.97)	(8.93 ± 3.40)	(13.8 ± 6.74)	(16.4 ± 7.91)	(13.6 ± 13.8)
Small intestine	23.2 ± 16.8	26.6 ± 22.8	33.0 ± 29.8	19.6 ± 7.49	18.0 ± 7.31
	(1.05 ± 0.616)	(1.32 ± 1.09)	(2.92 ± 1.55)	(4.45 ± 2.68)	(2.19 ± 2.04)
Large intestine	17.8 ± 20.8	10.5 ± 8.05	20.4 ± 19.5	10.0 ± 5.72	7.09 ± 3.72
	(1.00 ± 1.51)	(0.562 ± 0.528)	(1.52 ± 0.900)	(2.10 ± 1.23)	(0.769 ± 0.419)
Lung	35.0 ± 52.0	34.2 ± 16.9	27.5 ± 22.2	136 ± 48.4	58.1 ± 34.3
	(1.80 ± 2.69)	(1.70 ± 0.960)	(2.04 ± 1.03)	(27.6 ± 5.82)	(4.23 ± 1.99)
Heart	3.42 ± 2.51	2.97 ± 1.48	3.12 ± 1.48	3.03 ± 3.28	2.04 ± 0.558
	(0.157 ± 0.112)	(0.116 ± 0.0422)	(0.328 ± 0.133)	(0.563 ± 0.461)	(0.247 ± 0.177)
Fat	4.19 ± 1.81	4.57 ± 2.41	4.54 ± 2.34	8.30 ± 8.06	2.17 ± 1.51
	(0.190 ± 0.0649)	(0.228 ± 0.0934)	(0.586 ± 0.485)	(1.82 ± 1.99)	(0.315 ± 0.410)

Tissue	FBX-PG (n = 3)				
	**0.5 h**	**2 h**	**4 h**	**8 h**	**12 h**
Plasma	19.5 ± 4.96	36.1 ± 18.0	13.5 ± 4.33	6.55 ± 1.88	29.6 ± 13.5*
Liver	64.2 ± 26.7*	102 ± 22.8*	146 ± 83.4*	52.4 ± 5.07*	30.1 ± 6.06
	(3.33 ± 1.10)*	(3.31 ± 1.42)	(10.1 ± 6.52)*	(8.38 ± 2.00)*	(1.17 ± 0.507)*
Kidney	22.0 ± 8.32*	14.7 ± 8.09	10.3 ± 1.48	13.9 ± 6.55	11.8 ± 4.09
	(1.11 ± 0.220)*	(0.396 ± 0.0366)	(0.845 ± 0.421)	(2.03 ± 0.558)	(0.477 ± 0.264)
Stomach	159 ± 48.3	126 ± 40.1	234 ± 86.7	197 ± 156	74.1 ± 30.3
	(9.05 ± 5.52)	(4.36 ± 2.82)	(17.2 ± 1.05)	(26.5 ± 19.8)	(3.02 ± 2.08)
Small intestine	21.1 ± 3.72	32.9 ± 18.3	21.0 ± 2.44	20.9 ± 1.31	13.8 ± 5.30
	(1.10 ± 0.117)	(0.989 ± 0.385)	(1.65 ± 0.442)	(3.43 ± 1.29)	(0.530 ± 0.270)
Large intestine	17.3 ± 7.20	28.5 ± 24.2	49.7 ± 15.9	21.7 ± 19.6	16.1 ± 9.72
	(0.970 ± 0.564)	(1.40 ± 1.88)	(4.25 ± 2.86)	(3.00 ± 2.20)	(0.513 ± 0.186)
Lung	311 ± 209*	121 ± 55.6*	144 ± 75.9*	178 ± 63.5	121 ± 85.3
	(15.8 ± 8.37)*	(3.85 ± 1.78)	(10.4 ± 4.38)*	(27.0 ± 3.81)	(4.14 ± 2.23)
Heart	2.24 ± 0.511	3.95 ± 2.01	2.45 ± 0.978	2.45 ± 2.47	1.71 ± 1.09
	(0.118 ± 0.0270)	(0.111 ± 0.0134)	(0.180 ± 0.0229)	(0.350 ± 0.282)	(0.0589 ± 0.0276)
Fat	3.23 ± 0.964	4.30 ± 1.29	3.81 ± 1.11	6.10 ± 4.48	1.58 ± 0.489
	(0.167 ± 0.0340)	(0.174 ± 0.167)	(0.286 ± 0.0328)	(0.988 ± 0.680)	(0.0599 ± 0.227)

* Statistically different (P-value < 0.05) from FBX mice.
